# Exploring the identity of individual plant cells in space and time

**DOI:** 10.1111/nph.19153

**Published:** 2023-07-22

**Authors:** Marina Oliva, Ryan Lister

**Affiliations:** ^1^ ARC Centre of Excellence in Plant Energy Biology, School of Molecular Sciences University of Western Australia Perth WA 6009 Australia; ^2^ The Harry Perkins Institute of Medical Research, QEII Medical Centre and Centre for Medical Research The University of Western Australia Perth WA 6009 Australia

**Keywords:** cellular responses to stimuli, developmental cell trajectories, plant cell identity, regulatory networks, single‐cell genomics, spatial transcriptomics

## Abstract

In recent years, single‐cell genomics, coupled to imaging techniques, have become the state‐of‐the‐art approach for characterising biological systems. In plant sciences, a variety of tissues and species have been profiled, providing an enormous quantity of data on cell identity at an unprecedented resolution, but what biological insights can be gained from such data sets? Using recently published studies in plant sciences, we will highlight how single‐cell technologies have enabled a better comprehension of tissue organisation, cell fate dynamics in development or in response to various stimuli, as well as identifying key transcriptional regulators of cell identity. We discuss the limitations and technical hurdles to overcome, as well as future directions, and the promising use of single‐cell omics to understand, predict, and manipulate plant development and physiology.

**Table:** 

	Contents	
	Summary	61
I.	Introduction	61
II.	Generating atlases of cell identities and their markers	62
III.	Identifying co‐expression networks and their putative regulators	62
IV.	Studying the dynamics of cell fates	63
V.	Precise phenotyping of plants	64
VI.	Conclusion and perspectives	64
	Acknowledgements	65
	References	66

## I. Introduction

Biologists have a long history of creating classifications to resolve the irreducible complexity of biological systems. In multicellular organisms, multiple criteria have been successively used to categorise cells into cell ‘types’ with a specific signature: morphology, ontogeny, function, position within a tissue or the expression of specific sets of marker genes. Each component sheds light on particular aspects of cell identity, but none is sufficient to describe the diversity of cell states, which also show dynamic acclimation in response to diverse factors. This is particularly true for plants, which exhibit extensive postembryonic plasticity, have cells that can deviate from their developmental fate, and are affected by various environmental cues.

The recent and rapid development of single‐cell omics technologies offers an opportunity to overcome limitations of previous classifications and obtain precise unbiased high‐dimensional snapshots of cell diversity, where each cell can ultimately be in a different state. Here, we will review recent developments in understanding plant cellular identity at single‐cell resolution, focussing on biological insights they have brought to plant sciences, and discuss future directions for the field.

## II. Generating atlases of cell identities and their markers

Since the first plant single‐cell RNA‐seq analyses were pioneered by manually harvesting fluorescently labelled cells (Brennecke *et al*., 2013; Efroni *et al*., 2015), plant single‐cell studies have increased considerably in number and scale. Droplet‐based technologies that allow transcriptome (i.e. single‐cell RNA‐seq, scRNA‐seq) and chromatin accessibility (i.e. single‐cell ATAC‐seq, scATAC‐seq) profiling of thousands of individual cells or nuclei (Box 1) simultaneously has facilitated exploration of cell identities in numerous plant tissues including roots, leaves (Liu *et al*., 2020, 2022; Kim *et al*., 2021; Lopez‐Anido *et al*., 2021; Apelt *et al*., 2022; Procko *et al*., 2022), shoot meristems (Satterlee *et al*., 2020; Zhang *et al*., 2021b; Conde *et al*., 2022), stems (Chen *et al*., 2021; Xie *et al*., 2022; Du *et al*., 2023; Tung *et al*., 2023), pollen (Nelms & Walbot, 2019), female gametophytes (Song *et al*., 2020), callus (Zhai & Xu, 2021) and nodules (Cervantes‐Pérez *et al*., 2022; Wang *et al*., 2022; Liu *et al*., 2023), and in different plant species: Arabidopsis, maize (Satterlee *et al*., 2020; Marand *et al*., 2021; Xu *et al*., 2021), moss (Kubo *et al*., 2019), poplar (Chen *et al*., 2021; Conde *et al*., 2022; Xie *et al*., 2022; Du *et al*., 2023; Tung *et al*., 2023), rice (Liu *et al*., 2021; Zhang *et al*., 2021a), *Catharanthus roseus* (Sun *et al*., 2022), sorghum and millet (Guillotin *et al*., 2023), tomato (Omary *et al*., 2022), and tobacco (Kang *et al*., 2022).

Box 1 **Capturing cells: biases and limitations**

Most single‐cell analyses in plants have relied on the generation of protoplasts by enzymatic digestion of cell walls, a process that has some limitations: it induces cell type‐specific changes in gene expression, cannot be used for tissues or species that are recalcitrant to digestion, and favours the selection of easily dissociable cell types. Although some studies have addressed these limitations by, for example, excluding genes affected by protoplasting from analysis and focussing on tissues that are readily dissociated by protoplasting, others have opted for the isolation of nuclei rather than cells.

Nuclei isolation is a much faster process and allows profiling of tissues that cannot be digested. However, single‐nucleus RNA‐seq often results in data sets with lower complexity. A recent study compared single‐cell and single‐nucleus profiles of Arabidopsis and maize roots and showed that, while both globally capture the same biological patterns, nuclei profiles detected 1.4–2.7 times fewer transcripts compared to cells (Guillotin *et al*., 2023). Consequently, nuclear data sets tend to generate fewer clusters and can struggle to differentiate closely related or subcellular identities. To obtain an equivalent number of clusters as protoplasts, twice as many nuclei are required (Guillotin *et al*., 2023).

Another important consideration is the capture of rare cell populations, which poses challenges in both single‐cell and single‐nuclei assays. Many studies aim to capture the emergence of specific cell identities or transient cellular states, which often represent a small fraction of the total cells. Methods for enriching specific and low abundance identities, such as fluorescence‐activated cell sorting (Roszak *et al*., 2021; Serrano‐Ron *et al*., 2021; Omary *et al*., 2022), dissection of tissues (Gala *et al*., 2021; Omary *et al*., 2022), or removal of overrepresented cells (Kim *et al*., 2021) have played a crucial role in compensating for biases in cell proportions caused by isolation approaches and improving the resolution of cell trajectories.

In each data set, the initial step is to classify cells into clusters of similar expression patterns that represent cell ‘types’. Differential expression tests are then used to find cluster‐specific marker genes, aiding cluster annotation when prior knowledge of tissue expression patterns is available. However, for many plant species or tissues, this information is lacking or requires extensive literature searches to determine the necessary number of marker genes for reliable cluster annotation. Computed markers are extremely valuable for identifying genes highly specific to known or new cell populations, some of which are of unknown function, opening new avenues of investigation. For example, Gala *et al*. (2021) characterised transcriptional differences in a small population of xylem pole pericycle (XPP) cells that give rise to lateral root primordia (LRP), identifying genes specifically expressed in early LRP formation. By testing their functionality using an XPP‐specific dCas9‐driven repressor system, the authors demonstrate how candidate gene identification using scRNA‐seq enabled discovery of new regulators of LRP development (Gala *et al*., 2021). Interestingly, profiling of maize ears showed that SNPs in or within 2 kb of scRNA‐seq marker genes are more likely to be associated with yield traits, and revealed associations with ear traits that were not found by bulk RNA‐seq (Xu *et al*., 2021), suggesting a unique application of scRNA‐seq data to improve crop traits.

Markers can also be used to map different cell clusters *in vivo* when prior knowledge is limited, using reporter lines or *in situ* hybridisation, enabling generation of precise gene expression atlases. Recently, plant biologists have taken advantage of the rapidly progressing field of spatial transcriptomics to directly measure targeted (Laureyns *et al*., 2021; Nobori *et al*., 2023) or untargeted (Xia *et al*., 2022; Liu *et al*., 2023) transcript abundance *in situ*, removing the need of a two‐step approach. To date, most plant spatial transcriptomics studies have lacked cellular resolution, but new spatial technologies such as MERFISH (Chen *et al*., 2015) that achieve cellular‐ or subcellular resolution will allow the generation of powerful atlases of cell identities in the near future.

Single cell atlases are also extremely valuable for understanding complex physiological processes involving the coordination of various cell types. Sun *et al*. (2022) used scRNA‐seq to study the spatial regulation of the biosynthesis of monoterpenoid indole alkaloids (MIAs) in *Catharanthus roseus* leaves, a diverse class of metabolites of great pharmaceutical importance. The authors showed that the MIA biosynthetic pathway is partitioned into three distinct cell types and identified several transporters that could play central roles in shuttling MIA intermediates between inter‐ and intracellular compartments (Sun *et al*., 2022). These transporters are promising targets for future metabolic engineering to modulate MIA yield *in vivo*.

Overall, single‐cell technologies are enabling a precise molecular portrait of cell identities, where identification of new cell state‐specific features can open the way to targeted functional studies.

## III. Identifying co‐expression networks and their putative regulators

Gene co‐expression networks characterise related genes based on shared expression profile, revealing functional and biological relationships between genes, including co‐regulation. While network analyses from bulk RNA‐seq samples can be biased by cell type heterogeneity, scRNA‐seq provides an unparalleled opportunity to understand how gene–gene relationships shape cell identity. Zhai & Xu (2021) leveraged single‐cell co‐expression analyses to identify common targets of WUSCHEL RELATED HOMEOBOX 5, PLETHORA 1 and 2, markers of the pluripotent middle cell layer of Arabidopsis calli. They demonstrated that these transcription factors (TF) promote auxin production via the upregulation of *TRYPTOPHAN AMINOTRANSFERASE OF ARABIDOPSIS 1*, an essential step for pluripotency acquisition during regeneration from calli (Zhai & Xu, 2021).

These approaches are particularly powerful when coupled with transcription factor binding site information, such as DAP‐seq (O'Malley *et al*., 2016) or ChIP‐seq, to infer cell type‐specific gene regulatory networks, as recently demonstrated (Ferrari *et al*., 2022). Co‐expression of a TF and its putative targets can be used as a proxy for cell autonomous activity and to build regulatory networks, but co‐expression of target genes of a TF in another cell type can indicate potential non‐cell autonomous regulation. Wendrich *et al*. (2020) intersected root scRNA‐seq profiles with known target genes of the vascular TARGET OF MONOPTEROS 5/LONESOME HIGHWAY (TMO5/LHW) TF complex and found that, surprisingly, a large proportion of their target genes are expressed in root hair cells, far from their expression domain. They demonstrated that, under low‐phosphate conditions, TMO5/LHW triggers cytokinin biosynthesis, which diffuses to direct epidermal cell size and fate, improving the root's efficiency to forage phosphate (Wendrich *et al*., 2020).

Since TFs require or actively promote open chromatin to bind their target sequences, assays of chromatin accessibility are as crucial as TF expression to understand the establishment of cell type‐specific transcriptional programs. Accessible chromatin region (ACR) profiling in single nuclei across various maize organs and cultivars showed that most ACRs are cluster‐specific, associated with cell type‐specific marker genes, and have a lower density of polymorphisms, but are more associated with phenotypic variation, suggesting a key role of chromatin accessibility and its regulation in driving developmental programs (Marand *et al*., 2021). In both maize and Arabidopsis, co‐embedding of scRNA‐seq and snATAC‐seq data has been used to predict cell type‐specific regulatory TFs by associating an increase in ACRs at sites containing a TF binding motif with an increase in expression of the cognate TF (Dorrity *et al*., 2021; Marand *et al*., 2021).

In sum, gene co‐expression modules inferred from scRNA‐seq data are a powerful tool to infer gene function and identify putative regulators and their networks, especially when combined with TF binding data sets or co‐embedded with snATAC‐seq data. Although the integration of scRNA‐seq and snATAC‐seq data is still challenging, the development and optimisation of single cell co‐assays that profile multiple modalities simultaneously will help establish a clearer link between chromatin accessibility and expression, in addition to other epigenetic features such as histone modifications (Box 2).

Box 2 **Towards multi‐omics profiles**

Gene expression is regulated through an intricate interplay of various mechanisms, including epigenetics and chromatin accessibility. While scRNA‐seq provides crucial information about cell types/states and their dynamics, it cannot directly capture information about the underlying regulatory mechanisms that govern gene expression. On the contrary, snATAC‐seq measures the regional accessibility of chromatin within individual nuclei, which can reflect regulatory regions and potential gene regulatory elements. By combining scRNA‐seq and snATAC‐seq data, a more comprehensive understanding of the regulatory landscape governing gene expression at the single‐cell level can be gained.

While some concordant patterns of chromatin accessibility and expression have helped identify regulatory events in roots of different plant species, as explained in Section III, globally chromatin accessibility seems to be more variable than gene expression (Dorrity *et al*., 2021; Farmer *et al*., 2021; Marand *et al*., 2021). This is partially due to the association of chromatin accessibility with other regulatory mechanisms, such as gene silencing (Marand *et al*., 2021), but also to technical artefacts (e.g. different sensitivities, biases, noise levels, normalisation methods), making it difficult to uncover the regulatory landscape. Although computational methods to integrate datasets across modalities have been developed, their performance is difficult to assess and validate experimentally (Argelaguet *et al*., 2021).

Recently, droplet‐based methods that capture both chromatin accessibility and gene expression in thousands of cells simultaneously, such as the 10× Genomics Multiome assay or ISSAAC‐seq (Xu *et al*., 2022), or combinatorial methods such as SHARE‐Seq (Ma *et al*., 2020), have been developed and successfully used in animal systems. While these methods have not been optimised and reported for plant nuclei yet, they will certainly help gain a better understanding of the role of chromatin accessibility in the control of gene expression in the coming years.

## IV. Studying the dynamics of cell fates

Cellular heterogeneity captured by scRNA‐seq can also be used to recapitulate a continuous spectrum of states rather than a discrete classification. Computational methods have been developed to reconstruct trajectories by ordering cells based on their gene expression similarities, and assigning to the cells a *pseudotime* that symbolises the degree of change overtime, resulting in topologies that can vary from a simple differentiation path to complex trees. Plants are ideally suited for such analyses: they contain niches of pluripotent cells that can give rise to multiple cell fates along differentiation gradients that cannot be finely explored by bulk methods. Multiple studies have used root tip scRNA‐seq to investigate temporal regulation of specific (Roszak *et al*., 2021) or multiple differentiation lineages simultaneously in Arabidopsis (Denyer *et al*., 2019; Jean‐Baptiste *et al*., 2019; Ryu *et al*., 2019; Shulse *et al*., 2019; Zhang *et al*., 2019; Wendrich *et al*., 2020; Liu *et al*., 2021; Shahan *et al*., 2022) or rice (Zhang *et al*., 2019; Liu *et al*., 2021).

Initial enrichment of cell subpopulations based on reporter expression, followed by scRNA‐seq, can enable high‐resolution analysis of specific cell lineages. For example, Lopez‐Anido *et al*. (2021) used fluorescence‐activated sorted cells to reconstruct stomatal lineage trajectories and demonstrated that, contrary to the prevailing paradigm of stomata differentiation requiring sequential expression of the three regulators *SPEECHLESS* (*SPCH*), *MUTE* and *FAMA*, the *SPCH* expression domain overlaps with *MUTE* and *FAMA*. By selectively downregulating *SPCH* using artificial miRNAs driven by the *MUTE* promoter, they showed that this late‐stage expression of *SPCH* is required to maintain fate commitment of guard mother cells, revealing new roles for this known regulator (Lopez‐Anido *et al*., 2021).

While in the above examples, all stages of the trajectories could be extracted from the same sample, some studies require collection of samples at different time points. For instance, Omary *et al*. (2022) characterised the ontogeny of tomato shoot‐borne roots by profiling their phloem‐associated tissue of origin and three subsequent developmental stages. Trajectory reconstructions demonstrated that cells transition from their progenitor identity to the root stem cell and root cap fates, a transition defined by expression of an uncharacterised TF (*SHOOT BORNE ROOTLESS*) that is also expressed in tomato roots. Knocking out this single gene was sufficient to inhibit formation of shoot borne roots, demonstrating the power of single‐cell trajectory analyses in identifying key regulators of transient cell states (Omary *et al*., 2022).

In summary, trajectory analyses are a powerful approach for identifying transient cell states and regulators of cell fate commitment. However, these transient, and hence rare, populations and their associated complex trajectory events require a sufficient number of cells to be detected; thus, trajectory analyses will benefit from increasing numbers of cells profiled in new studies, and using alternate methods to compensate for bias in cell proportion (Box 1).

## V. Precise phenotyping of plants

The fine‐grained portrait of cell identities provided by single‐cell omics approaches offers a unique opportunity to detect subtle phenotypes that could not easily be detected by other techniques, including well‐studied mutants such as *scarecrow* (*scr*). This mutant is well known for lacking an asymmetric division in the ground tissue, resulting in a single layer with a mixed identity. However, a detailed understanding of how the cortex and endodermis identities are distributed in this mutant layer remained elusive until recently. Shahan *et al*. (2022) used a wild‐type scRNA‐seq root atlas to annotate *scr* cells, and pseudotime analysis of all cells assigned as cortex or endodermis revealed that, in the absence of SCR, the ground tissue transitions from a cortex‐like state to an endodermal state during differentiation. In another study, Graeff *et al*. (2021) used scRNA‐seq profiling of *bri1 brl1 brl3* triple mutant roots to correlate their reduced cell anisotropy with downregulation of arabinogalactan‐encoding genes, suggesting a mechanism by which brassinosteroid signalling controls root anisotropic cell expansion.

Single‐cell transcriptomics also enable the study of cell identity differences in related species, providing insights into plant cell type evolution. Profiling the developmental lineages of ray and fusiform cells, major xylem cell types, in the stem of four divergent woody angiosperms showed that, while ray lineages are highly conserved, fusiform lineages vary across core eudicots, basal eudicots and magnoliids (Tung *et al*., 2023). A comparative analysis of root scRNA‐seq of three grass species showed that cell types diverged at different rates, driven in part by co‐option of gene modules from one cell type to another (Guillotin *et al*., 2023). Notably, their analysis suggests that maize columella cells rapidly diverged by incorporating a gene module involved in mucilage biosynthesis from a putatively ancestral expression pattern in the cortex (Guillotin *et al*., 2023).

Cell molecular phenotyping can also be performed in response to treatments and changes in growth conditions. A time‐series scRNA‐seq analysis of brassinosteroid (BR) treatment response in Arabidopsis roots revealed that brassinosteroids trigger a shift from proliferation to elongation in cortex cells by inducing cell wall‐related genes, and identified two BR‐induced TFs specifically affecting the cortex trajectories. They functionally confirmed the role of these TFs in mediating BR‐induced cell elongation via the upregulation of cell wall‐related genes (Nolan *et al*., 2023). In another study profiling the Arabidopsis root after cytokinin (CK) treatment, the authors demonstrated that an increase in CK levels is counteracted by the upregulation of CYTOKININ OXIDASE 3 in procambium cells via the activation of the mobile TF SHORTROOT, to ensure well‐balanced CK levels (Yang *et al*., 2021).

These studies demonstrate the power of single‐cell approaches in characterising changes in cell identity in different genotypes and species, and in response to various perturbations, including the spatiotemporal control of hormonal responses.

## VI. Conclusion and perspectives

**Fig. 1:**
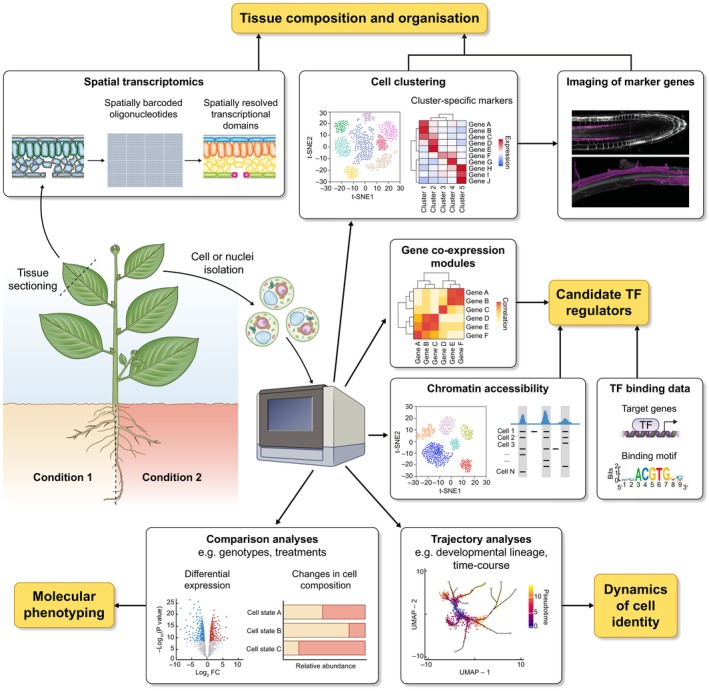
Biological insights gained from single‐cell genomics. Summary of the different single‐cell assays currently used in plant sciences, the associated computational analyses and their biological interpretation (orange boxes). TF, transcription factors. Created with BioRender.com.

The multidimensional information collected by single‐cell genomics has already provided a wealth of insights into the diversity of cell identities in many plant tissues, their transcriptional regulators, response to perturbations, and even conservation across species (Fig. 1). Increasing the number and scale of single cell studies, and advancing computational integration methods (Argelaguet *et al*., 2021), will be critical to improve the resolution and statistical significance of various analyses, such as detection and markers of rare cell states, cell fate trajectories, and changes in gene expression or cell state proportion in mutants or in response to stimuli.

While measuring the mRNA content of single cells has been extremely valuable for defining cell types and states, development of co‐assays that profile other molecules such as small RNAs or even proteins and metabolites, as well as different epigenetic features, will further refine our understanding of cell identities and reveal important biological insights. How well can cell identities be characterised with molecules other than mRNA, alone or in combination? How well does the epigenetic landscape correlate with mRNA‐defined cell states and how dynamic are they? It will be crucial for these methods, developed on animal tissues, to be optimised and applied to plant sciences. This also applies for spatial omics techniques, which have now been successfully used to detect other features such as chromatin accessibility (Deng *et al*., 2022) and proteins in 3D samples (Bhatia *et al*., 2022).

Another aspect that has not yet been explored in plant single‐cell studies is cell type‐specific natural variation in gene expression, and its genetic basis. scRNA‐seq offers enormous opportunities for mapping expression quantitative trait locus across different cell types and dynamic processes, many of which were obscured in bulk methods. Profiling a large pool of plant accessions, in a variety of contexts, will provide deep insights into the underlying cause of differential gene expression that determine different cell states, and perhaps the molecular basis of their responses to different natural environments.

Many cited studies in this review showcase the power of single‐cell analyses in inferring regulatory networks, offering valuable insights for functional studies. Beyond classical mutant analyses, plant biologists have recently developed a range of tools for precise spatial and temporal control of gene expression, and in response to a combination of inputs (Brophy *et al*., 2022; Lloyd *et al*., 2022; Guiziou *et al*., 2023). Combining the predicted regulatory elements identified by single‐cell genomics with synthetic biology toolkits will help reprogramming and modulating cell identities in plants, and could ultimately be used to boost plant and crop performance in different environments.

## Competing interests

None declared.
